# Optimization of *trans*-Splicing for Huntington's Disease RNA Therapy

**DOI:** 10.3389/fnins.2017.00544

**Published:** 2017-10-10

**Authors:** Hansjörg Rindt, Colton M. Tom, Christian L. Lorson, Virginia B. Mattis

**Affiliations:** ^1^Department of Veterinary Pathobiology, Bond Life Sciences Center, University of Missouri, Columbia, MO, United States; ^2^Cedars-Sinai Medical Center, Board of Governors Regenerative Medicine Institute, Los Angeles, CA, United States

**Keywords:** Huntington's disease, HD, *Huntingtin*, *HTT*, *trans*-splicing, therapy

## Abstract

Huntington's disease (HD) is a devastating neurodegenerative disorder caused by a polyglutamine (polyQ) expansion in exon 1 of the *Huntingtin* (*HTT*) gene. We have previously demonstrated that spliceosome-mediated *trans*-splicing is a viable molecular strategy to specifically reduce and repair mutant HTT (mtHTT). Here, the targeted tethering efficacy of the pre-mRNA *trans*-splicing modules (PTM) in HTT was optimized. Various PTMs that targeted the 3′ end of HTT intron 1 or the intron 1 branch point were shown *trans*-splice into an HTT mini-gene, as well as the endogenous HTT pre-mRNA. PTMs that specifically target the endogenous intron 1 branch point increased the *trans*-splicing efficacy from 1–5 to 10–15%. Furthermore, lentiviral expression of PTMs in a human HD patient iPSC-derived neural culture significantly reversed two previously established polyQ-length dependent phenotypes. These results suggest that pre-mRNA repair of mtHTT could hold therapeutic benefit and it demonstrates an alternative platform to correct the mRNA product produced by the mt*HTT* allele in the context of HD.

## Introduction

Huntington's Disease (HD) is an autosomal dominant neurodegenerative disorder caused by an expanded polyglutamine repeat (polyQ) in one allele of the *Huntingtin* (*HTT*) gene (The Huntington's Disease Collaborative Research Group, 1993). More than 35 polyQs causes disease, and the number of repeats is inversely correlated to the onset age and severity (Andrew et al., 1993; Duyao et al., 1993; The Huntington's Disease Collaborative Research Group, 1993). The disease affects ~6 per 100,000 in Caucasian populations (Pringsheim et al., 2012), with devastating consequences including progressive motor dysfunction, chorea, cognitive impairment, psychiatric abnormalities (Vonsattel et al., 2008), sleep disturbances (Arnulf et al., 2008), eventually leading to death (DiFiglia et al., 1995). These symptoms are due to the dysfunction and eventual death of specific projection neurons within the brain (Ross and Tabrizi, 2011). In particular, medium spiny neurons of the striatum, followed by the pyramidal neurons of the cortex, are most affected by disease. The loss of these neurons can occur years before overt clinical symptoms (Rosas et al., 2008; Aylward et al., 2011).

The *HTT* gene encodes a protein that is evolutionarily conserved, with homologs found as distant as the multicellular amoeba *Dictyostelium discoideum* (Eichinger et al., 2005). This degree of conservation implicates *HTT* as a necessary gene for the survival of multicellular organisms. Consistent with this notion, a *Htt*-null in murine models results in early embryonic lethality (e7.5) (Duyao et al., 1995; Nasir et al., 1995; Zeitlin et al., 1995). *HTT* is ubiquitously expressed and has roles in a multitude of cellular functions including protein transport (DiFiglia et al., 1995; Gutekunst et al., 1995; Block-Galarza et al., 1997), protein-protein interactions (Takano and Gusella, 2002), transcriptional regulation (Kegel et al., 2002; Sugars and Rubinsztein, 2003), inhibition of apoptosis (Rigamonti et al., 2000), and embryonic development (Duyao et al., 1995; Nasir et al., 1995; Zeitlin et al., 1995). While the expanded allele is associated with disease development, from an evolutionary perspective, the increasing CAG trinucleotide repeat length in *HTT* homologs correlates with an increase in brain evolution (Zuccato and Cattaneo, 2016). Potentially, the expanded repeat assists in the development of more complex nervous systems and that further pathogenic expansion of HD is an unintended side effect of this evolutionary process (Zuccato and Cattaneo, 2016). Like many other neurodegenerative diseases, it is currently unknown why this ubiquitously expressed protein specifically causes neuronal degeneration. There are a multitude of theories of the contribution of mutant HTT (mtHTT) protein to cell death including: specific mtHTT cleavages (Lunkes et al., 2002; Graham et al., 2006; Ratovitski et al., 2009) and modifications (Steffan et al., 2004; Gu et al., 2009; O'Rourke et al., 2013; Vicente Miranda et al., 2016), buildup of toxic aggregates (DiFiglia et al., 1997; Scherzinger et al., 1997) or soluble mtHTT oligomers (Takahashi et al., 2008; Leitman et al., 2013; Ramdzan et al., 2017), and haploinsufficiency of wild-type HTT (Humbert, 2010).

RNA splicing is the editing of nascent pre-mRNA, thereby producing mRNA by removing the intronic regions. Part of this highly dynamic and regulated process involves recognition of intronic regions by the spliceosomal complex, including: a “GU” donor site at the 5′ end of the intron, an “A” branchpoint near the 3′ end of the intron, and a poly-pyrimidine tract with an “AG” splice acceptor site at the 3′ end of the intron (Supplementary Figure 1). *cis-*Splicing occurs when introns are spliced out of a pre-mRNA and the flanking exons of the gene are ligated together. However, splicing can also occur between exons of two different RNA molecules, termed *trans-*splicing. *trans*-Splicing generates a chimeric mRNA and has been demonstrated to occur naturally in trypanosomes (Murphy et al., 1986; Sutton and Boothroyd, 1986), rat hepatocytes (Caudevilla et al., 1998), and even human cells (Flouriot et al., 2002; Wu et al., 2014). A technology predicated upon these naturally occurring *trans*-splicing events was developed in 1999, where an endogenous transcript was modified via spliceosome-mediated pre-mRNA *trans*-splicing to include an exogenous exon (Puttaraju et al., 1999). Previously, we have presented proof-of-principle experiments demonstrating that exon one replacement of *HTT* by *trans*-splicing can be achieved in cultured cells, both into a *HTT* mini-gene and the endogenous *HTT* pre-mRNA, suggesting a novel mechanism for mtHTT repair (Rindt et al., 2012).

These exogenous exons were termed pre-mRNA *trans-*splicing molecules (PTMs) and have three main modalities: a region that specifically binds to the target endogenous RNA, an artificial intron, and the exogenous “replacement” exon. Each sequence can be modified accordingly to improve efficacy and specificity to the *trans*-splicing event into the endogenous RNA (Garcia-Blanco, 2003). To further refine and increase the efficacy of HTT *trans*-splicing, a novel series of PTMs were designed to specifically bind to either the 5′ or 3′ ends of HTT intron 1. The coding sequence of each PTM-replaced exon 1 with a non-disease exon 1 sequence including 21 CAG trinucleotide repeats. PTMs that targeted the 3′ end of HTT intron 1 or the intron 1 branch point resulted in enhanced levels of *trans*-spliced RNA compared to the original HD *trans*-splicing RNA, demonstrating a correction of the HTT allele at the molecular level. To determine whether delivery of the optimized PTMs increased functional HTT protein, PTMs were expressed in a neural culture derived from HD patient induced pluripotent stem cells (iPSCs). In each instance PTM expression correlated with improvements in two previously established HTT CAG-expanded dependent phenotypes: susceptibility to BDNF withdrawal and ATP deficiency. Together, these results demonstrate that *trans*-splicing can be optimized to increase functional HTT protein, suggesting that *trans*-splicing could potentially serve as a therapy for HD.

## Materials and methods

### Cloning of constructs

#### HTT mini-gene construct

The HTT mini-gene has been previously described in Rindt et al. (2012). It contained exon 1 with 42 CAG repeats and exons 2–3, separated by intervening sequences. The sequences were based on Genbank accession number NT_006051. The two introns were shortened to 860 and 109 bp, respectively. The mini-gene was subcloned into pCI-neo (Promega, E1841) and expression was driven by the cytomegalovirus (CMV) promoter/enhancer.

#### PTM constructs

The PTM constructs consisted of three portions: (1) the replacement exon 1 of HTT with 21 CAG repeats, (2) the splicing domain with an U1 snRNP binding site at the 3′ end of exon 1 and a triplet repeat of an intronic splice enhancer (ISE), and (3) the tether which binds to intron 1 by antisense base pairing. The constructs were generated by custom gene synthesis (Geneart). The PTM was inserted behind the CMV promoter into pMU1 (Coady et al., 2008). pMU1 also contained an eGFP expression module expressed from a separate promoter. For viral delivery, the PTMs were inserted into the lentiviral vector pSIN18 (Gropp et al., 2003) at the EcoRV site, where they were expressed off of a CMV promoter. This vector also contains an EF1a promoter-driven GFP upstream of the PTM. All tether sequences are included in Supplementary Table 1.

#### Transient expression of PTMs

HEK293 cells were cultured in Dulbecco's Modified Eagle's Medium (DMEM; Gibco, 11965-092) containing high glucose and supplemented with 10% fetal bovine serum (Hyclone) and 100 U penicillin/100 μg streptomycin (Invitrogen) per mL. Cells were transiently transfected when they had reached ~90% confluency using PEI (Polysciences, 23966-1) or lipofectamine 2000 (Invitrogen, 12566014), according to the manufacturer's recommendations. Mini-gene and PTM plasmids were co-transfected at a 1:1 ratio. Empty vector controls were extensively performed, but never demonstrated a positive response (data not shown). Alternatively, the PTM plasmid was transfected by itself in experiments designed for *trans*-splicing of endogenous HTT pre-mRNA. Cells were harvested 24–48 h post-transfection.

#### RNA isolation and RT-PCR

RNA was isolated using Tri-Reagent (Sigma Aldrich, T9424) following the manufacturer's instructions. RNA was resuspended in 10 mM Tris–HCl pH 8.2, 1 mM EDTA, and concentrations were measured using a Nanodrop (Thermo Fisher). cDNA was synthesized using 1 μg of RNA and random primers following the SuperScript III protocol (Invitrogen, 18080-044). PCR was performed using two different procedures. For amplifications outside the HTT exon 1 CAG repeat and the adjacent GC-rich region, Pfu enzyme (prepared in-house) with Thermopol buffer (New England Biolabs, B9004S) was used. For amplification across the exon 1 CAG repeat, Taq PCRx with 2 × enhancer solution (Invitrogen, 11495-017) was used, per manufacturer instructions. Primer sequences are all included in Supplementary Table 1.

#### Generation, characterization, and propagation of iPSC lines

Human fibroblast lines were obtained from one HD patient with an expanded CAG *HTT* allele of 180 CAG repeats (CS97iHD180n) and from two non-HD “controls” with 33 (CS83iCTR33n) or 28 (CS14iCTR28n) repeat CAG alleles. Reprogramming was conducted by non-integrating methods, as previously described (Mattis et al., 2015). Neural progenitor aggregates were generated by manually lifting iPSC colonies from the feeder layers directly into 70:30 DMEM (Gibco, 11965-092):F12 (Gibco, 11765-054) plus 2% B27 without vitamin A (Gibco, 12587-010) supplemented 100 ng/mL basic fibroblast growth factor (bFGF, Peprotech, 100-18B), 100 ng/mL epidermal growth factor (EGF, Millipore, GF316), and 5 μg/mL heparin (Sigma Aldrich, H3393-50KU) in polyhema-coated flasks to prevent attachment, as previously described (Ebert et al., 2013). iPSC-derived neural progenitors were expanded as spherical aggregates and passaged weekly with a chopping technique (Svendsen et al., 1998).

#### Lentiviral generation and infection

Virus was produced by triple transfection of HEK293 FT cells with pSIN18-PTM, the helper plasmid psPAX2 (originally developed by D. Trono and obtained from Addgene #12260) and the envelope plasmid pVSV-G for pseudotyping. After 48 h, cell culture supernatant was collected and filtered through a 0.45-μm PES membrane, followed by centrifugation at 53,000 g for 90 min to pellet viral particles. Pellets were resuspended in phosphate-buffered saline (PBS) and stored at 4°C until use.

Neural progenitor spheres were infected with lentivirus encoding the PTMs by first allowing the spheres to settle in a flask, then carefully removing the majority of the media from the flask. Cells were then moved into a conical with TrypLE (Gibco, 12604-013) in order to dissociate the aggregates. After a 5 min incubation at 37°C, the spheres were washed and triturated into a single cell suspension. 3 × 10^6^ cells were infected in a total of 1 mL of conditioned media with 100 ng p24/ mL of virus. An additional 1 mL of fresh media was added the next day. The cells were given a complete refeed 3 days later.

Lentiviral generation and infection were carried out in accordance of the respective safety practices, equipment and facility requirements outlined by the Institutional Biosafety Committees at the authors' respective institutes.

#### iPSC striatal-like differentiations

iPSC colonies grown on Matrigel in TeSR media (feeder-free) were scraped into EGF/FGF2 (100 ng/mL each) containing media (70:30 DMEM:F12 plus 2% B27 without vitamin A) and grown as floating neural progenitor spheres for at least nine passages. Cells were then plated on PLO/laminin coated coverslips and differentiated in DMEM:F12 with 1% N2 (Gibco, 17502-048) (neural induction media; NIM) for 5 days. BDNF (20 ng/mL; Peprotech 450-02) was then added for 2 days. For the next 21 days cells were differentiated in 20 ng/mL BDNF, rhShh (200 ng/mL; R&D 1845-SH), and Dkk1 (100 ng/mL; R&D 1096-DK-010) to promote a rostral forebrain fate. Afterwards, cells were then matured in 20 ng/mL BDNF, dibutyryl cyclic AMP (dbcAMP, 0.5 mM; Sigma D0260) and valproic acid (VPA, 0.5 mM; Sigma P4546) for the rest of the differentiation (until day 42). Medium was half-changed twice per week, or as needed.

#### Immunocytochemistry

Neural progenitor spheres were plated on poly-lysine and laminin-coated coverslips from 24 h to 43 days (striatal differentiations) before being fixed in 3.2% paraformaldehyde (EMS). Cells were then permeabilized using 0.2% Triton X-100 in PBS for 10 min at room temperature before incubation with mouse monoclonal anti-HTT (Millipore MAB5374; 1:1,000), rabbit polyclonal anti-GFP (LifeTechnologies A11122; 1:1,000), and chicken anti-βIIITubulin/TUJ1 (Aves labs, TUJ; 1:200) overnight at 4°C and then washed in PBS. The slides were further incubated with Alexa Fluor 488-conjugated donkey anti-rabbit secondary antibody (Invitrogen, A21206; 1:500), Alexa Fluor 647-conjugated donkey anti-mouse secondary antibody (Invitrogen, A31571; 1:500), and Alexa Fluor 594-conjugated goat anti-chicken secondary antibody (Invitrogen, A11042; 1:500) for 60 min at room temperature, followed by PBS washes. After a 5 min incubation with 4′,6-diamidino-2-phenylindole (DAPI), slides were mounted with fluoromount (Sigma-Aldrich, F4680) and observed under fluorescence microscope (Leica).

#### Western blot

Neural progenitor sphere pellets were lysed by incubation in 20–40 μl SDP buffer (50 mM Tris pH 8.0, 150 mM NaCl, 1% Igepal, 0.1% SDS, 40 mM B-glycerophosphate (Sigma Aldrich, 251291), 10 mM NaF (Sigma Aldrich, S7920), 1X Roche complete protease inhibitor (Roche, 05892791 001) specifications. 40 to 100 μg of total protein was then resolved on 10% low-bis acrylamide gels [Resolving layer: 8% Acrylamide (BioRad 161-040), 0.04% BIS (BioRad, 161-1042), 0.375M Tris pH 8.8, 0.075% TEMED, 0.075% APS; Stacking layer: 4% Acrylamide-Bis 29:1 (BioRad, 161-0157), 0.156 M Tris pH 6.8, 0.075% TEMED, 0.075% APS] as in Mattis et al. (2015) or Mini-Protean TGX 4-15% gel (BioRad, 450-1085). In order to determine band size a ladder was loaded into the first lane (Biorad Precision Plus Protein ™ Dual Color Standard, 161-0374). Briefly, lysates were transferred onto PVDF membrane (BioRad Turbo transfer, 170-4157) for 7 min at 1.3 A. For ECL detection, the membrane was blocked in 6% dry non-fat milk in Tris-buffered saline plus 0.1% Tween 20 (Sigma-Aldrich, p9416; 1:1,000) for 1 h at room temperature, and then exposed to primary antibody against HTT (Millipore, MAB5374) in block for 1.5 h at room temperature. Anti-mouse secondary antibodies conjugated to peroxidase (Jackson Labs, 715-035-150; 1:10,000) was applied in block for 1 h at room temperature, followed by exposure to chemiluminescence kit (Super Signal West Femto Maximum Sensitivity Substrate, Thermo Scientific, 34095). A separate blot was run using the same lysate and probed for GFP (1:1,000) as above (secondary: Jackson labs, anti-rabbit 711-0350152; 1:10,000). Both blots were then stripped per manufacturer's instructions (Restore Western Stripping Buffer, ThermoFisher Scientific, 21059) and reprobed for rabbit anti-actin as a loading control (Sigma, D05060; 1:300) as above. For fluorescence detection, the membrane was blocked in Odyssey blocking buffer (LiCor, 927–50,000) for 1 h at room temperature. Primary antibodies were applied as above. Anti-mouse 680 (LiCor, 926–68,072; 1:10,000) or anti-rabbit 800 (LiCor, 926–32,213; 1:10,000) secondary antibodies were used for their respective primaries and detected on a LiCor Odyssey CLx.

#### Filter trap assay

Neural progenitor sphere pellets were lysed by incubation in 20–40 μl SDP buffer for 30 min on ice with vortexing every 5 min. Cellulose acetate membrane (Whatman, 10404180) was equilibrated in 0.1% SDS for at least 10 min. After dot blotter (BioRad, 170-6545) was assembled, 30 μg sample was diluted in 200 μL 2% SDS and boiled for 5 min. Samples were added to each well and vacuum was used to filter the samples through the membrane. After washing with 0.1% SDS, the membrane was blocked for 1 h in 5% milk before immunoblotting using a mouse anti-polyQ antibody (Sigma 3B5H10, P1874; 1:1,000), as above. Anti-mouse secondary antibodies conjugated to peroxidase (Jackson Labs, 715-005-150; 1:10,000) was applied in block for 1 h at room temperature, followed by exposure to chemiluminescence kit (Super Signal West Femto Maximum Sensitivity Substrate, Thermo Scientific, 34095).

#### Cell titer-glo assay

Relative intracellular ATP values in neural progenitor cell extracts were measured using the CellTiter-Glo Luminescent Assay (Promega, G7571) according to manufacturer's instructions, using 100 μl of Cell-Titer Glo reagent and 100 μl of media containing 9,000 cells. This was plated in 96 well plates and luminescence was measured after 10 min using an Envision system (ThermoScientific, 2104 Multilabel Reader). Data for two separate clones for the HD180 line (CS97iHD180n1 and CS97iHD180n3) were pooled and presented as “HD,” as they were not significantly different via one-way ANOVA. Two separate control lines were similarly pooled (CS83iCTR33n1 and CS14iCTR28n5).

#### BDNF withdrawal

iPSC-derived neural progenitors were differentiated toward a striatal fate for 42 days and then transferred into basic NIM or NIM plus 100 ng/mL BDNF for 24 h. dbcAMP and VPA were removed from the medium in the above experiments, as they increase endogenous BDNF transcription, but are not critical for cell survival (Bredy et al., 2007; Pruunsild et al., 2011). After 24 h, cells were fixed in 3.6% PFA. Effects of BDNF withdrawal were assessed by quantifying TUNEL incorporation per total DAPI-stained nuclei, according to manufacturer recommendations (ThermoFisher, Click-iT® TUNEL Alexa Fluor® 594 Imaging Assay, C10246), using Metamorph software. All experiments were performed at least three separate times. As in the Cell Titer-Glo Assay, the data for the two separate clones for the HD180 line (CS97iHD180n1 and CS97iHD180n3) or two separate control lines (CS83iCTR33n1 and CS14iCTR28n5) were respectively pooled, as they were not found significantly different via one-way ANOVA.

#### Statistical analyses

Group means were tested by one-way ANOVA, with *post-hoc* Bonferroni testing to adjust for multiple comparisons. All results are expressed as mean ± standard error of the mean. In the figures, asterisk indicate *p*-values as indicated (^*^p < 0.05, ^**^p < 0.01, and ^***^p < 0.001). All experiments were performed at least three times.

## Results

**Figure 1:**
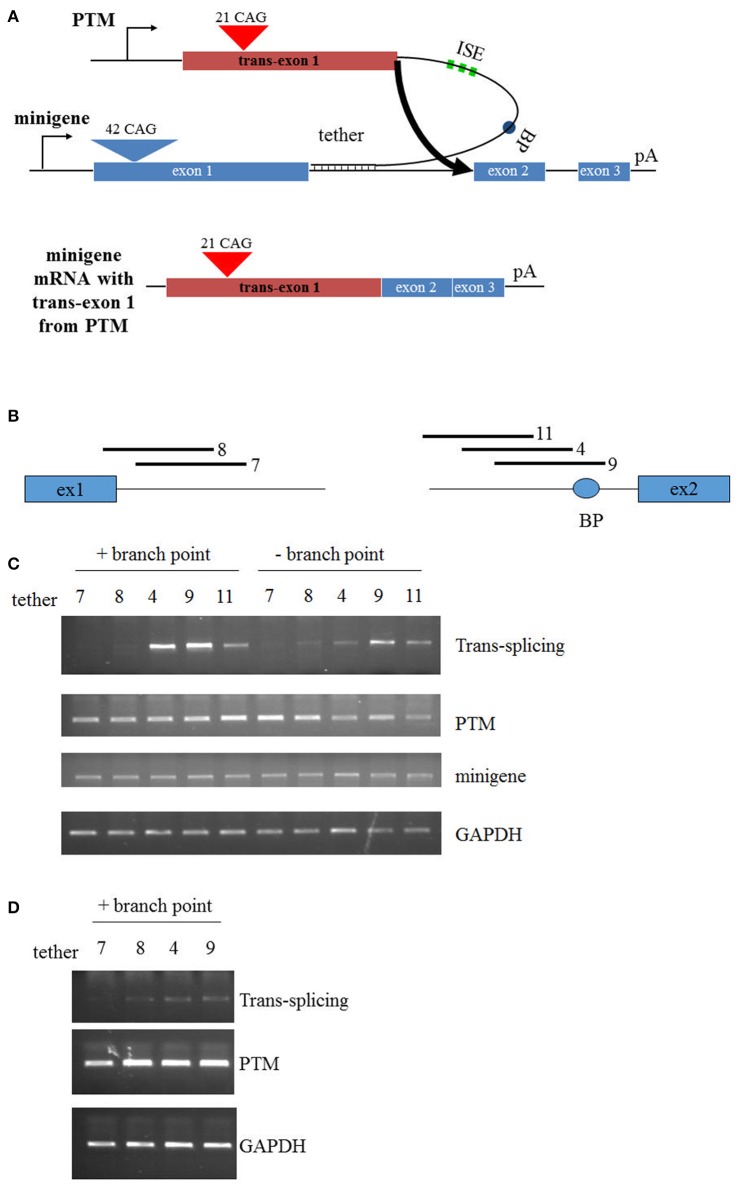
*Trans*-splicing in HEK293 cells. **(A)** Schematic of PTM targeting. The PTM is driven by the CMV promoter and contains 21 CAG repeats within HTT exon 1. The PTM contains intronic splice enhancers (ISE), a branch point (BP), and a “tether” that targets the PTM to specific sequences within the HTT or mini-gene pre-mRNA. The HTT mini-gene is driven by the CMV promoter and consists of exon1-2-3 with intervening introns and a polyA signal. **(B)** Relative position of the PTM tethering elements for the PTMs. T7 and T8 target the 5′ end of intron 1; T4, T9, and T11 target the 3′ end of intron 1. **(C)** Mini-gene *trans*-splicing in HEK293 cells. RT-PCR of total RNA isolated from HEK293 cells transiently transfected with the indicated PTMs and the HTT mini-gene. PTMs contained a splicing branch point (+branch point) or had the branch point removed with flanking restriction sites (−branch point). *trans*-splicing RNA, PTM expression, mini-gene expression, and endogenous GAPDH expression is shown. **(D)** Endogenous *trans*-splicing in HEK293 cells. RT-PCR of total RNA isolated from HEK293 cells transiently transfected with the indicated PTMs. *trans*-splicing RNA, PTM expression, and endogenous GAPDH expression is shown.

In the development of the original PTM that targeted replacement of HTT exon 1, few modifications were made to optimize the *trans*-splicing efficiency (Rindt et al., 2012). Therefore, the primary objective in this report was to enhance *trans*-splicing by targeting different regions within the HTT RNA. In this new series of PTMs, a non-disease encoding HTT exon 1 with 21 CAG repeats was fused to a synthetic stretch of RNA that functioned as the targeting sequence or the “tether” (Figure 1A). Additionally, to enhance splicing to the endogenous HTT pre-mRNA, three tandem repeats of a previously identified ISE element were incorporated into the PTM, as well as the HTT intron 1-derived branch point. PTMs were targeted to the 5′ or 3′ end of intron 1, with T9 overlapping the branch point upstream of exon 2 (Figure 1B). To initially examine the efficiency of *trans*-splicing, a previously developed mini-gene system was utilized that comprises a sub-genomic fragment of the HTT gene spanning exons 1, 2, and 3, separated by shortened introns 1 and 2, respectively. Fourty-two CAG tandem repeats were engineered into the exon 1 mini-gene sequence. Following co-transfection of the mini-gene with the PTMs into HEK293 cells, RT-PCR was used to specifically detect the *trans*-splicing product (Figure 1C). As expected, the original T7 PTM, which tethered to the 5′ end of intron 1, resulted in low but detectible levels of *trans*-splicing. Similarly, low levels of *trans*-splicing were also observed using the similarly targeted T8 PTM. In contrast, T4 and T9, the PTMs that were proximally positioned to exon 2 and disrupted the *cis* branch point within the mini-gene pre-mRNA, resulted in greater levels of *trans*-splicing (Figure 1C). Site-specific mutagenesis of the PTM branch point reduced *trans*-splicing efficiency in each instance, highlighting the importance of intact and functional splicing signals within the PTMs.

Having identified lead *trans*-splicing candidates using the mini-gene system, the branchpoint-containing PTMs were examined in a more challenging context: *trans*-splicing to endogenous HTT. HEK293 cells were transfected with PTM-expressing vectors and RNA was subsequently collected and analyzed via RT-PCR. The T4 and T9 PTMs were the most consistently active in this assay, resulting in high levels (10–15%) of *trans*-splicing product in multiple independent experiments (Figure 1D). Collectively, these results identify the T4 and T9 PTMs as *trans*-splicing RNAs that lead to enhanced replacement of the expanded HTT allele in a mini-gene system and with endogenous HTT in HEK293 cells. These therefore will serve as lead candidates in the following experiments that examine *trans*-splicing in disease-relevant contexts.

### PTMs can be expressed in neural stem cells derived from HD iPSCs

**Figure 2:**
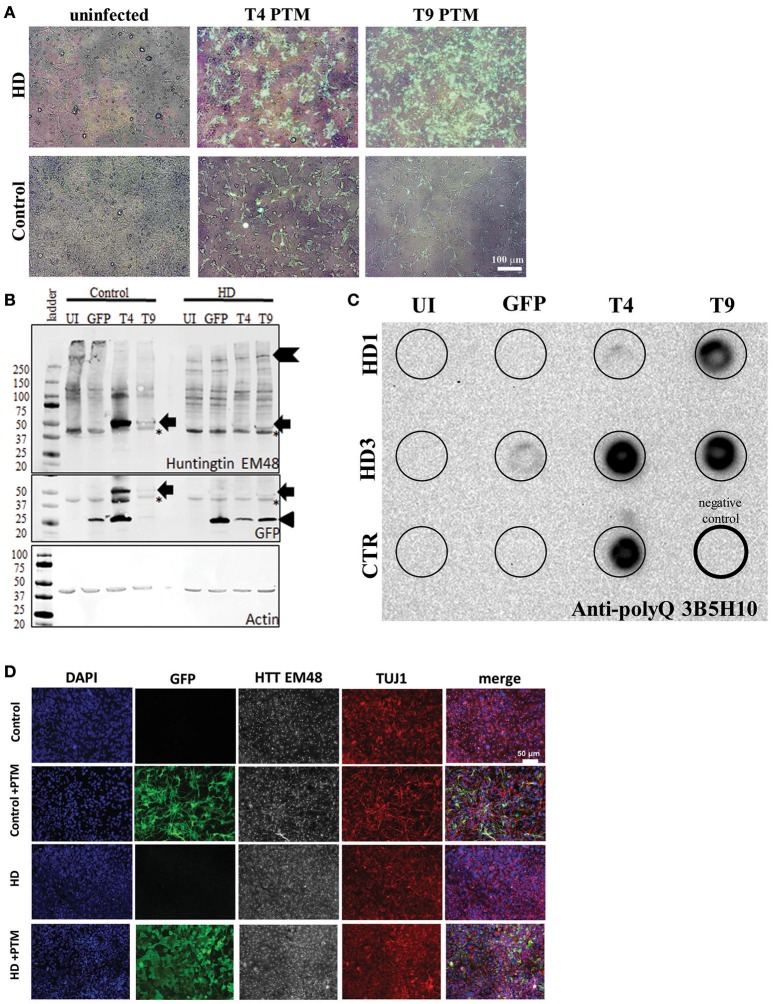
PTMs can be expressed in human HD iPSC-derived NPCs. **(A)** Overlays of GFP and bright-field live images of HD or control NPCs after infection with lentivirus. Uninfected cells do not express GFP. Cells infected with PTM-encoding lentivirus have GFP expression in the majority of cells. White scale bar length represents 100 μm. **(B)** HD and control iPSC-derived NPCs infected with PTM-encoding lentivirus express the predicted high molecular weight HTT (indicated by chevron) as well as a specific low molecular weight N-terminal HTT fragment (indicated by arrow). Actin was used as an internal loading control. HD and control iPSC-derived NPCs infected with PTM-encoding lentivirus express two detectible isoforms of GFP via Western blotting. The lower molecular weight band corresponds to the expected size for GFP (indicated by arrowhead), whereas the higher molecular weight band is at the same size as the band found in the PTM-expressing cells via an anti-HTT antibody (indicated by arrow). Huntingtin (EM48) gave an additional a non-specific band (found in both + and – PTM) around 37 kDa (^*^ indicates non-specific bands). **(C)** Filter trap assay detects that HD and control iPSC-derived NPCs infected with PTM-encoding lentivirus have more polyQ aggregation. The lower right lane (thick outside border) was loaded with lysis buffer only to serve as a negative control. **(D)** NPCs derived from HD and control iPSCs express similar levels and localization of HTT and β3-tubulin/TUJ1 (an immature neuron marker) regardless of presence of PTM (indicated by expression of GFP). White scale bar length represents 50 μm.

It has been previously demonstrated that PTMs can be used to successfully replace the mtHTT exon 1 in primary neural tissue from HD mice (Rindt et al., 2012). However, PTMs had yet to be expressed in human HD neural tissues. Therefore, the two PTMs identified with the greatest *trans*-splicing activity (T4 and T9) were over-expressed in neural progenitors derived from HD or control iPSC-derived neural progenitors (Ebert et al., 2013) via lentiviral infection. Post lentiviral infection, transgene incorporation into the progenitors was confirmed via live GFP expression, which was seen in the majority of cells (Figure 2A).

Next, HTT protein expression in the iPSC-derived neural progenitors was examined. First, Western blot analysis was performed in order to evaluate if reduction in endogenous mtHTT protein levels and/or the expression of the PTMs could be detected in the HD180 cells. As previously reported (Miller et al., 2011), the HTT antibody used to probe the blot preferentially recognize the CAG-expanded HTT protein expressed by the HD180 samples (EM48; Figure 2B, Supplementary Figure 2). While no significant decrease was seen in the high molecular weight band of mtHTT in the presence of the PTM (~350 kDa, chevron), there was an additional band detected (~47 kDa) in all samples overexpressing a PTM (Figure 2B, arrow). Interestingly, when probing the blot using an antibody that recognizes GFP, in addition to detecting a band at the predicted 27 kDa (Figure 2C, arrow-head), a band of similar size to the low molecular weight HTT isoform (~47 kDa) was also detected in samples expressing a PTM [Figure 2B (arrow), Supplementary Figure 2F]. In the GFP-infected alone cells only the predicted 27 kDa band was detected. One possible explanation of a band of this size would be that in addition to PTM *trans*-splicing to endogenous HTT, there may also be some fusion or *cis*-splicing within the PTM transgene to generate a GFP:PTM product.

To further examine the HTT protein produced by cells expressing the PTM, lysates were analyzed for aggregate formation via a filter trap assay. Protein lysate from PTM-expressing iPSC-derived neural cultures was filtered through a membrane that specifically traps and retains large protein aggregates, while small species, including protein monomers, pass through. As previously described, the HD iPSC-derived neural progenitors had little to no HTT aggregation detected (The HD iPSC Consortium, 2012). However, using an antibody specific to polyQ repeats, it was demonstrated that all lines overexpressing the PTMs had increased polyQ-containing aggregate retention (Figure 2C).

To determine if PTM expression induced the production of visibly detectable HTT aggregates, the EM48 *HTT* antibody was used for immunocytochemistry on iPSC-derived neural progenitors. HTT aggregates were not large enough to be detected, nor was there a different sub-cellular localization between the HD or control progenitors, regardless of the absence/presence of PTMs or GFP alone (Figure 2D). Therefore, these data demonstrate that while aggregates can be detected via extremely sensitive measures, they are not large enough to be considered a visible polyQ inclusion.

### PTMs demonstrate HD phenotypic reversal in neural cultures from HD iPSCs

A common concern for trans-splicing reactions relates to translational fidelity and the production of functional protein following the trans-splicing reaction. To determine whether functional HTT was produced following trans-splicing, two HD clones (180 CAG repeats) and two control iPSC-derived neural progenitor lines (33 or 28 CAG repeats) expressing the T4 or T9 PTMs were examined for HD phenotypic reversal. Uninfected cells, or those infected with a lentivirus encoding GFP alone, were used as a control.

**Figure 3:**
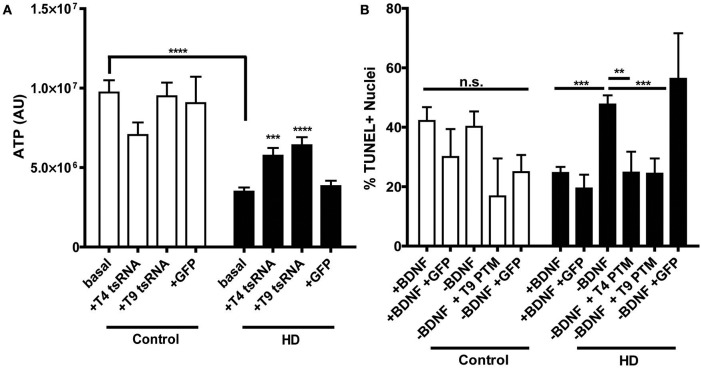
PTM expression in HD180 iPSC-derived neural cultures reverses established mtHTT phenotypes. **(A)** ATP levels in HD and control NPCs. Cells were dissociated and ATP levels were assayed in equal numbers of cells. Control NPCs had significantly more ATP than HD NPCs. However, expression of PTMs significantly increased ATP levels in the HD, but not control, NPCs. Expression of GFP did not significantly affect ATP levels. ^***^p < 0.001, ^****^p < 0.0001, one-way ANOVA. Data are plotted as means and SEM. At least three separate passages of NPCs were examined. **(B)** HD and control iPSCs, with or without PTMs, were differentiated toward a striatal fate for 42 days before BDNF was withdrawn for 24 h. Cells were fixed, assayed for TUNEL, and counter stained with Hoechst. The percentage of TUNEL-positive nuclei was calculated as a measure of toxicity. The HD180 iPSC-derived striatal cells showed more cell death after BDNF withdrawal than did control iPSC-derived striatal cells. However, BDNF withdrawal in the presence of PTMs, but not GFP, reversed this toxicity. ^**^p < 0.01, ^***^p < 0.001, one-way ANOVA. Data are plotted as means and SEM. At least three fields per coverslip (three coverslips per experiment) were counted at random during at least three separate differentiation experiments.

HD iPSC-derived neural progenitors derived have previously been shown to have a decreased metabolic rate (An et al., 2012; The HD iPSC Consortium, 2012; Ring et al., 2015), consistent with the energy deficiencies observed in HD patients (Mochel and Haller, 2011). Therefore, in order to establish whether PTM expression has an effect on this established HD phenotype, ATP levels were examined in the progenitors. As anticipated, both clones of the uninfected HD180 iPSC-derived neural progenitors demonstrated significantly lower ATP levels than the unaffected control neural progenitors (Figure 3A, Supplementary Figure 3). Expression of either the T4 or T9 PTM in the HD180 progenitors significantly increased ATP levels, compared to ATP levels in untreated cells. Expression of GFP alone did not impact ATP levels in the HD180 progenitor cells.

As the primary brain region affected in HD is the striatum, its generation is of particular interest for HD iPSC modeling. It has been previously demonstrated iPSC-derived HD striatal-like cultures exhibit a CAG-length dependent susceptibility to withdrawal of Brain Derived Neurotropic Factor (BDNF), resulting in a cell death phenotype (An et al., 2012; The HD iPSC Consortium, 2012; Lu et al., 2014; Mattis et al., 2015). To determine whether PTM expression could correct this, cell death was monitored in the HD iPSC-derived striatal cultures following a 24 h withdrawal of BDNF, after 42 days of differentiation. As each cell line has different basal levels of cell death, which is not HD-related, cell death after withdrawal with or without PTMs was compared within each cell line to the with BDNF condition. As expected, the control iPSC-derived cultures did not have increased cell death after withdrawal, regardless of the presence/absence of PTMs or GFP alone (Figure 3B). As previously described, the HD180 iPSC-derived cultures had significantly increased cell death after a 24 h withdrawal of BDNF, which was not affected by the expression of GFP alone. However, in the presence of either the T4 or T9 PTM, the amount of BDNF withdrawal-dependent cell death in HD180 lines was rescued, as determined by significantly reduced cell death in PTM-treated cultures. Collectively, these data indicated that the expression of a PTM for exon 1 mtHTT replacement via trans-splicing is a potential mechanism to reduce the well-defined HD pathology in iPSC-derived neural cultures.

## Discussion

This study provides further validation for the use of *trans-*splicing as a powerful potential therapy tool for genetic disorders such as HD. In this report, the objective was to determine whether targeting the PTM tether to different regions within the HTT pre-mRNA would lead to increased *trans*-splicing activity. In a variety of disease-relevant experimental contexts, targeting the *cis* branch point within the HTT pre-mRNA improved efficiency of the PTM in a variety of disease contexts.

As HD is a monogenic disorder, gene-based correction of mtHTT is a compelling prospective therapeutic tool. There are several paradigms for “gene therapy,” each with important benefits as well as potential drawbacks (for a complete review please see Keiser et al., 2016; Yang et al., 2016). RNA interference (RNAi) and antisense oligonucleotides (ASOs) are single-stranded nucleotide sequences that work by binding specifically to the target mRNA, thereby knocking down gene expression. ASOs in particular do hold great promise as a treatment for HD. They have been demonstrated to efficiently knock-down mtHTT in an allele-specific manner (Østergaard et al., 2013; Skotte et al., 2014; Southwell et al., 2014) and are currently in HD patient clinical trials (Rollnik, 2017). One drawback however, includes the inability of ASOs to cross the blood-brain-barrier, therefore requiring direct administration to the brain, and the need for repeatedly administration over the course of a lifetime, as they are not made in the cells. Short-hairpin RNAs and microRNAs act in a similar fashion to RNAi and ASOs to knock-down gene expression, but can be virally delivered. This allows for continued gene expression after a single dose, however dosage control is more challenging. Another method to reduce/eliminate mtHTT expression at the protein level is the use of virally-delivered intracellular antibodies (intrabodies). However, proper intrabody folding and low solubility in the reducing cytoplasmic environment are issues of functionality and stability for this proposed therapy (Biocca et al., 1995). New technologies have emerged in the past decade to alter gene expression or even permanently edit the mutant genes themselves, including zinc finger nucleases, transcriptional activator-like effector nucleases (TALENs), or clustered regulatory interspaced short palindromic repeat/CRISPR associated protein 9 (CRISPR/Cas9) (for review see Merkert and Martin, 2016). While these technologies can lead to the genetic correction of mtHTT at the DNA level, the current *in vivo* technology is limited, primarily due to the relatively low efficiency of genetic correction and the need to correct the expanded allele in many tissues throughout the body (Kolli et al., 2017; Yang et al., 2017). Future studies utilizing these technologies will hopefully continue to improve upon what is already demonstrated to be promising techniques, the current limitations lead us to optimize the strategy of *trans*-splicing as a potential addition to the therapy toolkit for HD.

*Trans-*splicing also has its own set of strengths and weaknesses. One positive attribute is the inherent control of gene expression: *trans*-splicing cannot lead to gene overexpression, since the maximal amount of the *trans*-splicing event relies upon the endogenous HTT promoter. This is important in terms of appropriate gene “dosing.” A temporary knock-down of all HTT (both mutant and wild-type), termed a “*HTT* holiday” (Lu and Yang, 2012), has been shown to be tolerated in animals (McBride et al., 2011) and to reverse some symptoms of disease (Boudreau et al., 2009; Drouet et al., 2009; Kordasiewicz et al., 2012). However, other disease contexts demonstrate the complexity of HD, since a 50% decrease in murine Htt expression induces behavioral and cognitive abnormalities in ~1/3 of the mice, coupled with neurodegeneration in adulthood (Nasir et al., 1995; O'Kusky et al., 1999). Additional studies have demonstrated that targeted inactivation of Htt in the adult mouse brain results in neurodegeneration, further demonstrating that a delicate balance is required when envisioning HD therapeutics (Dragatsis et al., 2000). These studies would implicate the need for balance between reducing mtHTT and maintaining some functional level of wildtype HTT. However, studies using a cre-lox system to deplete neural (using the Nestin or CamKII promoters) Htt expression in adult mice (>4 months) demonstrated no changes in total brain volume or expression of neuronal proteins (Wang et al., 2016). With these conflicting studies there is obviously still work to be done examining the necessity of HTT expression in the adult brain. *trans-*Splicing avoids the haploinsufficiency issues as the molecular reaction converts mtHTT to wildtype HTT and any off-target *trans*-splicing to the non-pathogenic allele still results in wildtype HTT expression. While this report does not address the potential of allele-specific targeting, the net effect of non-allele specificity is a steady-state level of HTT expression, with at least some mutant to wild-type HTT conversion. Ideally, in the future, further modifications could be made to allow for allele-specific *trans*-splicing by targeting the tethering region to SNPs in the mtHTT, as has been done for HD ASO therapy (Skotte et al., 2014). In moving forward toward *in vivo* studies the CMV promoter would ideally be replaced with something that has shown sustained and robust *in vivo* expression within the CNS, such as the CBA promoter (Gray et al., 2011).

Perhaps one of the most significant hurdles to *trans*-splicing involves *in vivo* efficiency (Berger et al., 2016). While efficiencies have been reported ranging from <1 to ~40%, here we demonstrate that the efficacy of *trans*-splicing to the HTT mini-gene is ~10–15%. However, as wildtype HTT has been demonstrated to have an anti-apoptotic effect (Rigamonti et al., 2000, 2001; Gervais et al., 2002), the small reduction in mtHTT, paired with the increase in corrected HTT, may be sufficient to confer a therapeutic benefit. Consistent with this notion, *trans*-splicing mediated induction of HTT protein lead to the phenotypic reversal within neural cells derived from HD patient iPSCs. Another potential problem lies with recent reports of toxic RNA mtHTT species, in addition to the toxic protein (Bañez Coronel et al., 2012, 2015). It is currently unknown what effect *trans*-splicing would have on these transcripts. Ideally, the endogenous exon 1 pre-mRNA that was not spliced into the mature mRNA would be targeted for degradation, a likely path as this transcript does not possess a polyA signal and should be rapidly degraded through normal cellular processes. However, if there were additional toxic transcripts produced from the trinucleotide repeat (Bañez Coronel et al., 2012, 2015), these products would most likely not be targeted by this technology. Lastly, there are potential issues of non-disease gene disruption via off-target effects of the PTM or integration of the viral cassette into the genome. This is a consideration that will need to be examined for all gene therapy paradigms in the future. Therefore, in moving forward toward a translational product, the PTMs would most likely need to use a non-integrating viral vector, such as AAV. While direct administration of virus to the CNS is an option, using a viral vector able to cross the blood-brain-barrier after peripheral administration would be ideal (Deverman et al., 2016). To date, *trans*-splicing to spurious genomic sites has not been detected in our hands, however, a variety of “-omics” platforms, including RNA- and whole genome sequencing should be utilized before advancing further toward the clinic.

One interesting finding in this study was a ~47 kDa band, detected via both a GFP and a HTT antibody. This potential GFP-HTT fusion product was only detected in iPSC-derived neural progenitors that expressed PTMs. Theoretically, the reversal of the energy and cell death phenotypes seen in the HD iPSC-derived neural progenitors or striatal cultures, respectively, is due to the exon 1 replacement of mtHTT with the PTMs. However, we cannot exclude the possibility that the PTM expression of an exogenous HTT exon 1 fused with GFP is not contributing to the phenotype reversal. While it is known that HTT has pro-survival roles, the speculation presented here that a non-CAG expanded exon 1 alone could be of benefit in the presence of mtHTT in its self is extremely interesting and worthy of future pursuit as a potential avenue of therapy.

*Trans*-Splicing has been proposed for a potential therapeutic agent in other genetic disorders (Supplementary Table 2). These studies have all demonstrated efficacy of *trans-*splicing *in vitro*, and some *in vivo* (for a complete review please see Berger et al., 2016). Presently, this study builds upon our previous publication (Rindt et al., 2012) to demonstrate that modification of the binding modality of the PTM increases *trans*-splicing efficacy from 1–5% (Rindt et al., 2012) to 10–15% by altering the tethering region to specifically target the branch point of the endogenous intron 1. One interesting finding of this study is the increased aggregate retention seen via filter-trap assay, possibly as a result of the N-terminal exon 1 fragment seen via Western blotting analyses. This increased aggregate retention was seen in all samples expressing a PTM, to varying degrees which likely correlate with the original infection efficiency. This increase in aggregate retention did not result in increased aggregate formation or localization seen via immunocytochemistry. While historically HTT aggregate formation has been thought of as pathogenic (Khoshnan et al., 2002; Colby et al., 2004; Takeuchi et al., 2014), more recent papers have questioned this paradigm (Arrasate et al., 2004). Evidence for each side exists. For example, aggregate-suppressing therapeutic candidates can suppress toxicity (Warrick et al., 1999; Heiser et al., 2000; Lecerf et al., 2001; Kazantsev et al., 2002; Yoshida et al., 2002; Wang et al., 2005). Alternatively, many cell types that do not undergo degeneration (Kuemmerle et al., 1999) express even more aggregates than those that do (Vonsattel et al., 1985). Additionally, the soluble HTT population has instead been implicated in disease pathology (Arrasate et al., 2004). A recent study demonstrates that the aggregation of mtHTT may be protective against apoptosis, but may ultimately lead to a slow eventual cellular necrosis (Ramdzan et al., 2017). The findings presented here from the reversal of known patient iPSC-derived neural phenotypes potentially support the hypothesis that increased aggregation may be protective in HD, at least in the short-term. Longer-term studies of the effect of *trans*-splicing PTMs as a potential HD therapeutic, including those in mouse models, will therefore need to be performed.

## Ethics statement

The iPSC lines used in this study were approved by the IRB/SCRO at CSMC. All fibroblasts have been de-identified for sharing with the research community prior to generation of iPSCs, and no personal health information is available to any investigators beyond those who were involved in depositing them.

## Author contributions

HR, CT, CL, and VM had substantial contributions to the conception or design of the work, the acquisition, analysis, or interpretation of data for the work. HR, CT, CL, and VM all drafted the manuscript and/or contributed critical revisions for important intellectual content and approved the final version. HR, CT, CL, and VM agree to be accountable for all aspects of the work in ensuring that questions related to the accuracy or integrity of any part of the work are appropriately investigated and resolved.

### Conflict of interest statement

The authors declare that the research was conducted in the absence of any commercial or financial relationships that could be construed as a potential conflict of interest. The reviewer UB and handling Editor declared their shared affiliation.

## Supplementary material

Supplementary Figure 1 Schematic outlining both *cis*- and *trans*-RNA splicing. Introns (lines) are removed from the pre-mRNA by the spliceosomal complex, including: a “GU” donor site at the 5′ end of the intron; an “A” branchpoint near the 3′ end of the intron; and a poly-pyrimidine tract with an “AG” splice acceptor site at the 3′ end of the intron. *cis-*Splicing occurs when introns are spliced out of a pre-mRNA and the two flanking exons (blue boxes) of the gene are ligated together. *trans-*Splicing occurs when exons from two different genes [or a gene and a PTM (red box)] are ligated together. In this schematic *trans-*splicing is represented by the PTM bound to the intron via the tether region which recognizes the intron sequence surrounding the branch point.

Supplementary Figure 2 Supplemental Western blots of PTM expression in NSCs. **(A–D)** HD and control iPSC-derived NPCs infected with PTM-encoding lentivirus express the predicted high molecular weight HTT (~350 kDa), as well as a specific low molecular weight N-terminal HTT fragment (indicated by arrow; anti-HTT EM48 antibody). Actin was used as an internal loading control. Huntingtin (EM48) blots picked up a non-specific band (found in both + and – PTM) around 37 kDa. **(E)** Quantification of ~47 kDa band. Bands were quantified in the linear range and graphed as a ratio of detected intensity (AU) to the intensity of the UI cells within the same HD or control. **(F)** HD and control iPSC-derived NPCs infected with PTM-encoding lentivirus express two detectible isoforms of GFP via Western blotting. The lower molecular weight band corresponds to the expected size for GFP (indicated by arrowhead), whereas the higher molecular weight band is at the same size as the band found in the PTM-expressing cells via an anti-HTT antibody (indicated by arrow). Actin was used as an internal loading control.

Supplementary Figure 3 PTM expression in HD180 iPSC-derived neural cultures reverses established mtHTT ATP phenotypes. ATP levels, expressed relative to the basal levels of control cells, in HD and control NPCs. Cells were dissociated and ATP levels were assayed in equal numbers of cells. Control NPCs had significantly more ATP than HD NPCs. However, expression of PTMs significantly increased ATP levels in the HD, but not control, NPCs. Expression of GFP did not significantly affect ATP levels. ^***^*p* < 0.001, ^****^*p* < 0.0001, one-way ANOVA. Data are plotted as means and SEM. At least at least three separate passages of NPCs were examined.
