# Supplementary figures and images for: Optimization of trans-Splicing for Huntington's Disease RNA Therapy
Source: Front Neurosci. 2017 Oct 10;11:544. doi: 10.3389/fnins.2017.00544 (PMC5641306; doi:10.3389/fnins.2017.00544)

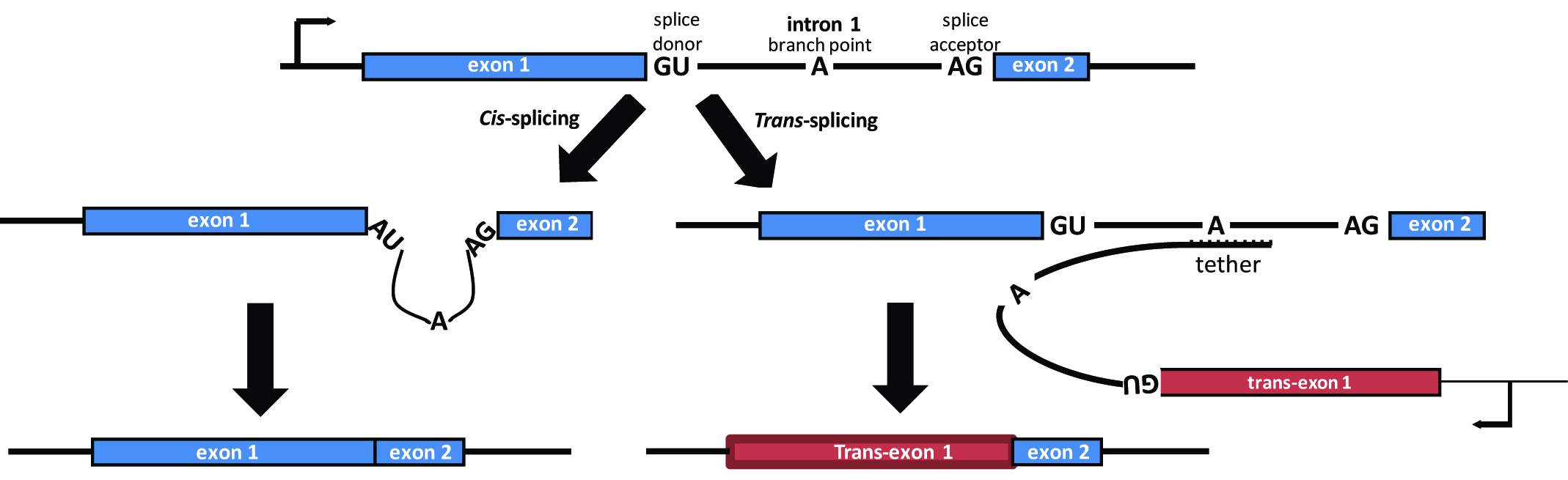

Supplement: Supplementary Figure 1 — Schematic outlining both cis- and trans-RNA splicing. Introns (lines) are removed from the pre-mRNA by the spliceosomal complex, including: a “GU” donor site at the 5′ end of the intron; an “A” branchpoint near the 3′ end of the intron; and a poly-pyrimidine tract with an “AG” splice acceptor site at the 3′ end of the intron. cis-Splicing occurs when introns are spliced out of a pre-mRNA and the two flanking exons (blue boxes) of the gene are ligated together. trans-Splicing occurs when exons from two different genes [or a gene and a PTM (red box)] are ligated together. In this schematic trans-splicing is represented by the PTM bound to the intron via the tether region which recognizes the intron sequence surrounding the branch point. [file Image1.jpg]

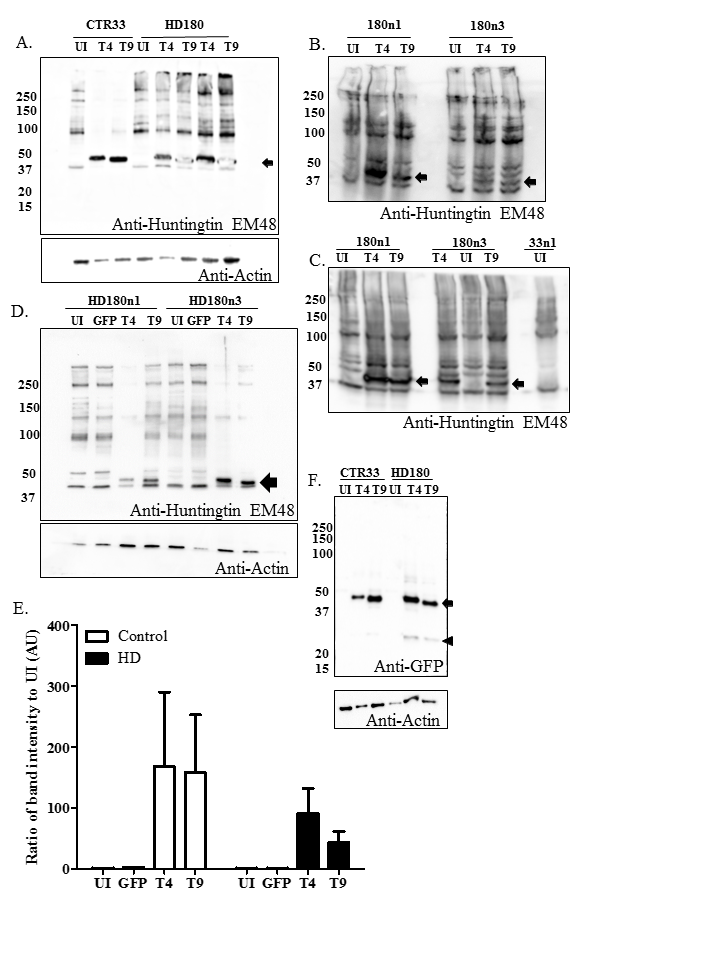

Supplement: Supplementary Figure 2 — Supplemental Western blots of PTM expression in NSCs. (A–D) HD and control iPSC-derived NPCs infected with PTM-encoding lentivirus express the predicted high molecular weight HTT (~350 kDa), as well as a specific low molecular weight N-terminal HTT fragment (indicated by arrow; anti-HTT EM48 antibody). Actin was used as an internal loading control. Huntingtin (EM48) blots picked up a non-specific band (found in both + and – PTM) around 37 kDa. (E) Quantification of ~47 kDa band. Bands were quantified in the linear range and graphed as a ratio of detected intensity (AU) to the intensity of the UI cells within the same HD or control. (F) HD and control iPSC-derived NPCs infected with PTM-encoding lentivirus express two detectible isoforms of GFP via Western blotting. The lower molecular weight band corresponds to the expected size for GFP (indicated by arrowhead), whereas the higher molecular weight band is at the same size as the band found in the PTM-expressing cells via an anti-HTT antibody (indicated by arrow). Actin was used as an internal loading control. [file Image2.TIF]

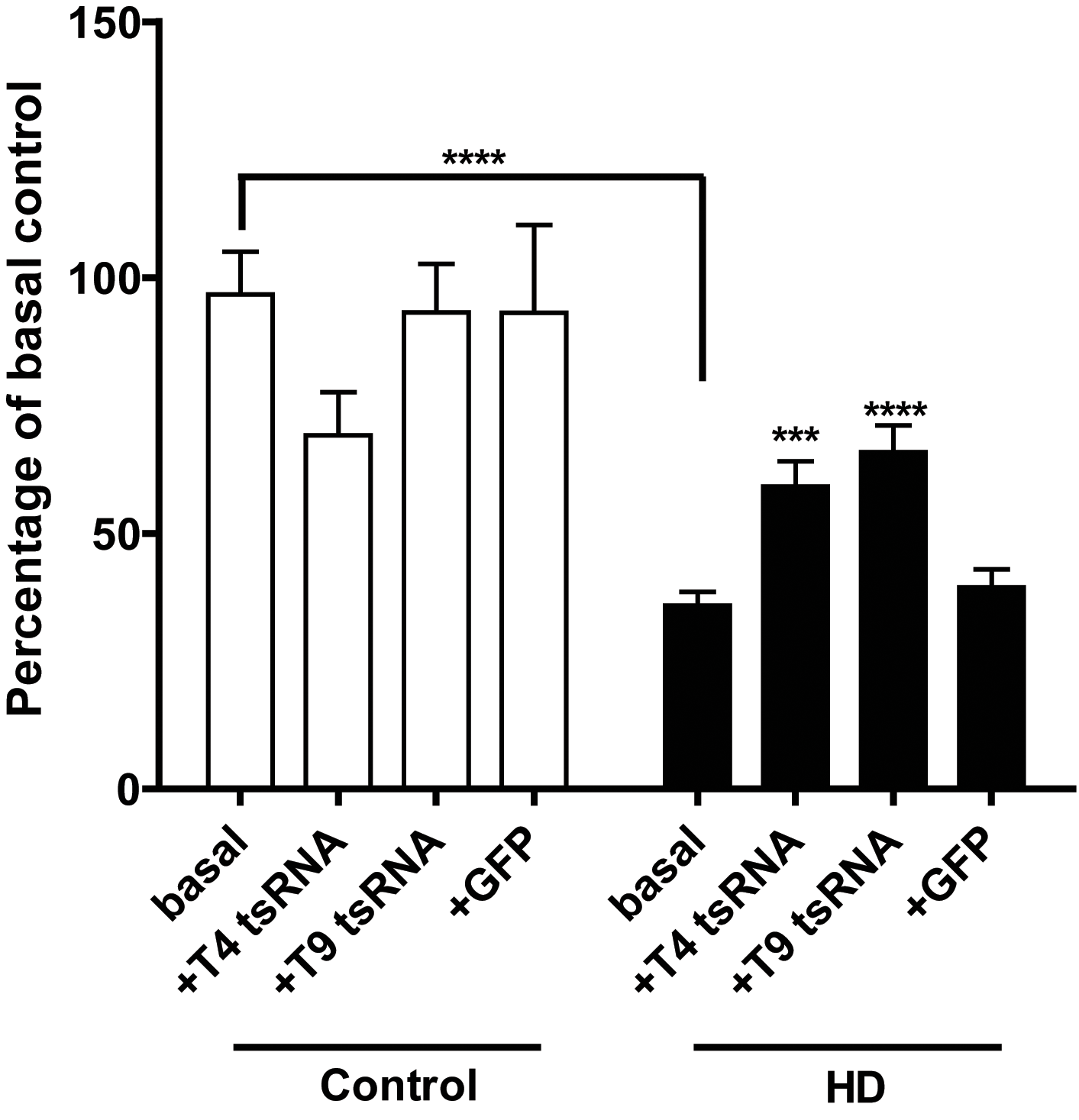

Supplement: Supplementary Figure 3 — PTM expression in HD180 iPSC-derived neural cultures reverses established mtHTT ATP phenotypes. ATP levels, expressed relative to the basal levels of control cells, in HD and control NPCs. Cells were dissociated and ATP levels were assayed in equal numbers of cells. Control NPCs had significantly more ATP than HD NPCs. However, expression of PTMs significantly increased ATP levels in the HD, but not control, NPCs. Expression of GFP did not significantly affect ATP levels. ***p < 0.001, ****p < 0.0001, one-way ANOVA. Data are plotted as means and SEM. At least at least three separate passages of NPCs were examined. [file Image3.TIF]
